# The usefulness of repeated CMR and FDG PET/CT in the diagnosis of patients with initial possible cardiac sarcoidosis

**DOI:** 10.1186/s13550-021-00870-y

**Published:** 2021-12-20

**Authors:** H. Mathijssen, T. W. H. Tjoeng, R. G. M. Keijsers, A. L. M. Bakker, F. Akdim, H. W. van Es, F. T. van Beek, M. V. Veltkamp, J. C. Grutters, M. C. Post

**Affiliations:** 1 Department of Cardiology, St. Antonius Hospital Nieuwegein, Koekoekslaan 1, 3435CM, Nieuwegein, Utrecht The Netherlands; 2 Department of Nuclear Medicine, St. Antonius Hospital Nieuwegein, Nieuwegein, Utrecht The Netherlands; 3 Department of Radiology, St. Antonius Hospital Nieuwegein, Nieuwegein, Utrecht The Netherlands; 4 Department of Pulmonology, St. Antonius Hospital Nieuwegein, Nieuwegein, Utrecht The Netherlands; 5 Department of Pulmonology, University Medical Centre Utrecht, Utrecht University, Utrecht, The Netherlands; 6 Department of Cardiology, University Medical Centre Utrecht, Utrecht University, Utrecht, The Netherlands

**Keywords:** Cardiac sarcoidosis, Diagnosis, Positron emission tomography, Cardiac magnetic resonance imaging

## Abstract

**Background:**

Cardiac sarcoidosis (CS) diagnosis is usually based on advanced imaging techniques and multidisciplinary evaluation. Diagnosis is classified as definite, probable, possible or unlikely. If diagnostic confidence remains uncertain, cardiac imaging can be repeated. The objective is to evaluate the usefulness of repeated cardiac magnetic resonance imaging (CMR) and fluorodeoxyglucose positron emission tomography (FDG PET/CT) for CS diagnosis in patients with an initial “possible” CS diagnosis.

**Methods:**

We performed a retrospective cohort study in 35 patients diagnosed with possible CS by our multidisciplinary team (MDT), who received repeated CMR and FDG PET/CT within 12 months after diagnosis. Imaging modalities were scored on abnormalities suggestive for CS and classified as CMR+/PET+, CMR+/PET−, CMR−/PET+ and CMR−/PET−. Primary endpoint was final MDT diagnosis of CS.

**Results:**

After re-evaluation, nine patients (25.7%) were reclassified as probable CS and 16 patients (45.7%) as unlikely CS. Two patients started immunosuppressive treatment after re-evaluation. At baseline, eleven patients (31.4%) showed late gadolinium enhancement (LGE) on CMR (CMR+) and 26 (74.3%) patients showed myocardial FDG-uptake (PET+). At re-evaluation, nine patients (25.7%) showed LGE (CMR+), while 16 patients (45.7%) showed myocardial FDG-uptake (PET+). When considering both imaging modalities together, 82.6% of patients with CMR−/PET+ at baseline were reclassified as possible or unlikely CS, while 36.4% of patients with CMR+ at baseline were reclassified as probable CS. Three patients with initial CMR−/PET+ showed LGE at re-evaluation.

**Conclusion:**

Repeated CMR and FDG PET/CT may be useful in establishing or rejecting CS diagnosis, when initial diagnosis is uncertain. However, clinical relevance has to be further determined.

**Supplementary Information:**

The online version contains supplementary material available at 10.1186/s13550-021-00870-y.

## Introduction

Sarcoidosis is a multisystem disease of unknown aetiology, characterized by non-caseating granulomas in multiple organs sometimes including the heart. About 5% of patients with systemic sarcoidosis have clinical evidence of cardiac sarcoidosis (CS), whereas autopsy and imaging studies suggest a higher prevalence around 20–30% [1–3]. Cardiac involvement is often non-specific and may range from asymptomatic to symptomatic conduction abnormalities, heart failure and sudden cardiac death [1, 4–6]. Considering the potential risk, early detection of cardiac involvement and appropriate treatment is of importance. However, the diagnosis of CS remains challenging due to the low sensitivity of endomyocardial biopsy, which is required for a “definite” diagnosis [4]. Therefore, diagnosis is usually based on advanced imaging techniques and multidisciplinary evaluation. In the St. Antonius Hospital, the diagnosis of CS is made by a multidisciplinary team (MDT) consisting of experienced cardiologists specialized in cardiac magnetic resonance imaging (CMR), pulmonologists and nuclear medicine physicians. The MDT classifies the diagnosis of CS as “probable” or “unlikely”. However, if no consensus can be reached, the diagnosis is classified as “possible” CS. In these patients, CMR and fluorodeoxyglucose positron emission tomography with computed tomography (FDG PET/CT) are repeated, in order to reject or establish a CS diagnosis by the MDT. The aim of this study was to evaluate the usefulness of repeated CMR and FDG PET/CT for the diagnosis of CS in patients who were initially diagnosed as “possible” CS.

## Methods

### Study design

A retrospective single centre cohort study was performed at the St. Antonius Hospital, a tertiary referral centre for sarcoidosis. Local institutional review board approval was obtained with a waiver of informed consent. All patients discussed in the CS MDT between January 2014 and March 2020 were evaluated. The diagnosis of CS in our MDT was based on the diagnostic criteria from the 2014 Heart Rhythm Society (HRS) consensus statement and 2016 Japanese Circulation Society (JCS) guideline [4, 7]. Before initial diagnosis, all patients received both CMR and FDG PET/CT. After multidisciplinary evaluation, the likelihood of CS was classified as “definite”, “probable”, “possible” or “unlikely”. When no consensus in the MDT could be reached, but imaging or clinical findings could be specific for CS (based on the 2014 HRS and 2016 JCS criteria), the diagnosis was deemed “possible”. These patients were re-assessed after 6–12 months with CMR and FDG PET/CT and included in the study. The variability between 6 and 12 months was based on logistical reasons and patient preference. After repeated imaging, patients were re-evaluated by the MDT and classified as either “probable”, “possible” or “unlikely”. Exclusion criteria included an interval between initial and repeated imaging > 12 months, insufficient imaging quality and suspected isolated CS. The primary outcome was the final CS diagnosis by the MDT after re-evaluation with CMR and FDG PET/CT. Secondary outcome parameters included change in immunosuppressive treatment, new cardiac symptoms, new/increased conduction abnormalities, ventricular arrhythmias, a decrease in left ventricle ejection fraction (LVEF) > 10% and all-cause mortality. Data were collected retrospectively by chart review. All data were stored in the web-based data manager REDCap.

### CMR and FDG PET/CT acquisition and analysis

All CMR images were acquired using a 1.5 T Philips MRI scanner with an eight-element phased-array cardiac coil. A vector electrocardiographic system was used for cardiac gating. A stack of short-axis cine slices of both the right- and left ventricle (8-mm thickness, no gap) from the base to the apex of the entire heart were acquired. If performed, T2-weighted short-tau inversion recovery images (indication myocardial oedema) with 8-mm slice thickness were acquired at short-axis orientation. Late gadolinium enhancement (LGE) images were obtained 12–20 min after intravenous administration of 0.4 ml/kg gadolinium. All CMR images were analysed by two experienced observers (F.A. and H. E.) blinded for clinical outcomes. The CMR images were scored on LVEF, increased T2-weighted signal, LGE and localization of LGE. Patients with abnormalities on CMR suggestive for CS were labelled as CMR+, while patients with no abnormalities suggestive for CS were labelled as CMR−.

FDG PET/CT examination was performed with a TF-64 combined PET/CT device (Philips Gemini, Medical systems, Eindhoven, The Netherlands). Patients were instructed to have a carbohydrate-restricted diet for 24 h followed by a fast of at least 6 h before injection of FDG. Dosage was based on body weight. 50 IE/kg unfractionated heparin was pre-administered intravenously to suppress physiologic uptake in the myocardium, with a maximum of 5000 IE. PET images were scored by a single experienced nuclear medicine physician (R.G.K.) for myocardial FDG uptake, localization and pattern. FDG uptake patterns were classified as: none, diffuse, focal and focal on diffuse. Maximum standardized uptake value (SUVmax) and normalized SUVmax (SUVmax divided by the SUVmean of the blood pool) were measured for all focal and focal on diffuse FDG uptake. The threshold for active inflammation was a SUVmax > 2.5 or a higher activity than the myocardial blood pool. SUVmax was measured at the active lesion. If no activity was present, SUVmax was measured at the basal interventricular septum. SUVmean was measured at the descending thoracic aorta at the level of the carina. Patients with myocardial uptake on FDG PET/CT, including a “diffuse” pattern were labelled as PET+. After CMR and FDG PET/CT analysis, four sub-groups were defined: CMR+/PET+, CMR+/PET−, CMR−/PET+ and CMR−/PET−.

### Statistical analysis

All statistical analyses were performed using SPSS statistics (version 26.0 for Windows; Armonk, NY: IBM Corp.). Continuous data were expressed as mean ± standard deviation or median [interquartile range]. Categorical data were reported as frequencies and percentages. Normality of data distribution was assessed using the Shapiro–Wilk test or Kolmogorov–Smirnoff test. The chi-squared test or Fisher’s Exact Test was used to compare categorical variables. The McNemar test was used to compare categorical variables of two related samples. The independent *t* test or One-way ANOVA was used to compare mean or median values of continuous variables. The paired samples *t* test or Wilcoxon signed rank test was used to compare means of two related samples. A two-tailed *p* value of < 0.05 was considered significant.

## Results

A total of thirty-five patients were included in this study. Table 1 summarizes the baseline characteristics. In total, 74.3% was male with a mean age of 52.5 ± 12.7 years. Extra-cardiac sarcoidosis was histologically or cytologically confirmed in 94.3%, while in 5.7% the diagnosis was based on clinical, laboratory and radiological findings [8]. Fourteen patients (40%) were already on immunosuppressive therapy for extracardiac sarcoidosis before the first MDT.

**Table 1:** Baseline characteristics

Variable	All patients (*n* = 35)
Age at diagnosis (years)	52.5 ± 12.7
Male sex	26 (74.3%)
Caucasian ethnicity	32 (91.4%)
Body mass index (m^2^/kg)	27.5 ± 3.7
Symptoms prior to first evaluation	
Chest pain	7 (20.0%)
Palpitations	17 (48.6%)
Syncope	3 (8.6%)
Dizziness	6 (17.1%)
NYHA functional class (I/II/III/IV)	12/18/5/0
Comorbidities	
Hypertension	9 (25.7%)
Diabetes mellitus	1 (2.9%)
Coronary artery disease	1 (2.9%)
Extra-cardiac sarcoidosis histologically or cytologically confirmed	33 (94.3%)
Extra-cardiac organ involvement	
Bilateral hilar lymphadenopathy	29 (82.9%)
Pulmonary	33 (94.3%)
Skin	1 (2.9%)
Neurologic	5 (14.3%)
Liver	3 (8.6%)
Ocular	5 (14.3%)
Laboratory results	
CRP (mg/L)	3.0 [2.0–4.5]
NT-proBNP (pg/mL) (*n* = 28)	44.0 [26.5–120.5]
ACE (U/L)	46.0 [33.0–68.0]
sIL-2R (pg/mL)	4057 [2887–5745]
Electrocardiogram results (*n* = 32)	
Sinus rhythm	31 (96.9%)
PQ-interval > 200 ms	4 (12.5%)
QRS duration (ms)	98.0 [91.0–112.0]
Left bundle branch block	0 (0.0%)
Right bundle branch block	4 (12.5%)
Left ventricular ejection fraction (%)	60.0 [55.0–62.0]
Immunosuppressive therapy at baseline	14 (40%)
Anti-arrhythmic drugs	6 (17.1%)
ACE-inhibitors or ARBs	11 (31.4%)

ACE, angiotensin-converting enzyme; ARB, angiotensin receptor blocker; CRP, C-reactive protein; NYHA, New York Heart Association; sIL-2R, soluble interleukin-2 receptor

### Primary outcome

**Fig. 1:**
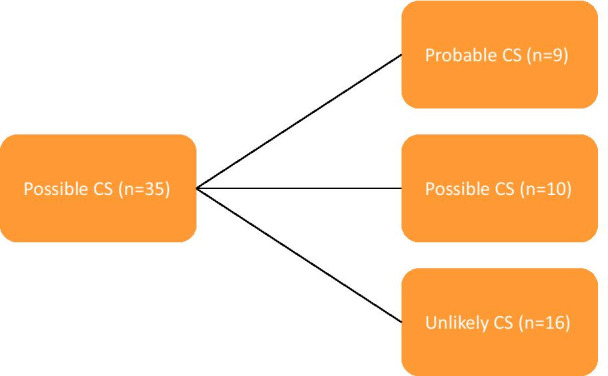
Reclassification of CS diagnosis after repeated imaging

Median time between both MDTs was 7.3 ± 2.1 months. In none of the patients repeated imaging was performed earlier due to clinical worsening. As shown in Fig. 1, twenty-five patients (71.4%) were reclassified after repeated imaging. Nine patients (25.7%) were reclassified as probable CS and sixteen patients (45.7%) as unlikely CS. Ten patients (28.6%) remained classified as possible CS. When using the 2014 HRS criteria or 2016 JCS criteria, 8 patients (22.9%) and 5 patients (14.3%) were diagnosed with probable CS, respectively.

### Imaging results

**Fig. 2:**
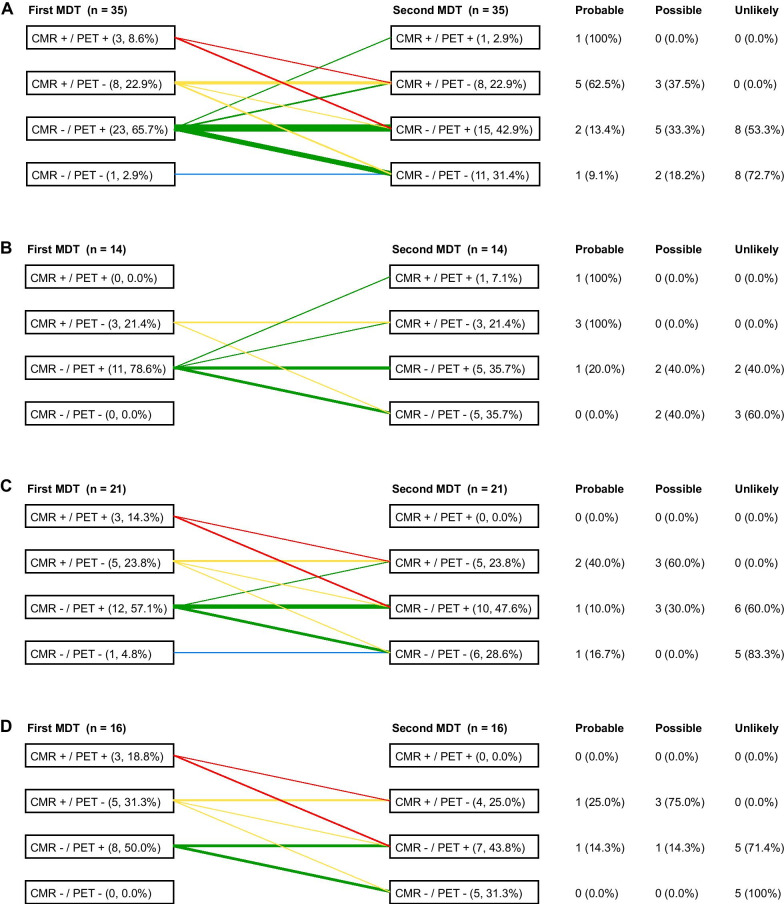
Imaging abnormalities at first and second MDT and corresponding final CS diagnosis in all patients (**A**), only patients with baseline immunosuppressive treatment (**B**), only patients without baseline immunosuppressive treatment (**C**) and only patients without baseline or newly started immunosuppressive treatment (**D**)

At baseline, eleven patients (31.4%) showed LGE on CMR. No patients showed increased T2-weighted signal at baseline, which was determined in 29 patients (82.9%). Myocardial FDG uptake was detected in twenty-six patients (74.3%), of whom ten (28.6%) showed a diffuse FDG uptake pattern, six (17.1%) showed a focal on diffuse FDG uptake pattern and ten patients (28.6%) showed a focal FDG uptake pattern (Table 2). Focal myocardial FDG-uptake was seen in the anterior (*n* = 2), antero-septal (*n* = 3), infero-septal (*n* = 2), inferior (*n* = 1), infero-lateral (*n* = 4), antero-lateral (*n* = 2) and apico-lateral wall (*n* = 1). When taking both imaging modalities into account, the majority of patients (65.7%) were classified as CMR−/PET+ at baseline, while one patient (2.9%) showed no abnormalities on cardiac imaging (CMR−/PET−) (Fig. 2A). This patient with histologically confirmed extra-cardiac sarcoidosis showed a second-degree AVB; however, this patient was also using beta-blockers which could have caused the AVB and was therefore classified as possible CS.

**Table 2:** FDG PET/CT results at baseline and re-evaluation

	Baseline (*n* = 35)	Re-evaluation (*n* = 35)	*p* value
Myocardial FDG uptake pattern			
Focal	10 (28.6%)	7 (20.0%)	0.51
Focal on diffuse	6 (17.1%)	4 (11.4%)	0.63
Diffuse	10 (28.6%)	5 (14.3%)	0.13
None	9 (25.7%)	19 (54.3%)	< 0.01
Cardiac SUVmax	4.2 [2.2–5.8]	1.8 [1.1–4.1]	< 0.01

FDG PET/CT, fluorodeoxyglucose positron emission tomography with computed tomography; SUVmax, maximum standardized uptake value

**Fig. 3:**
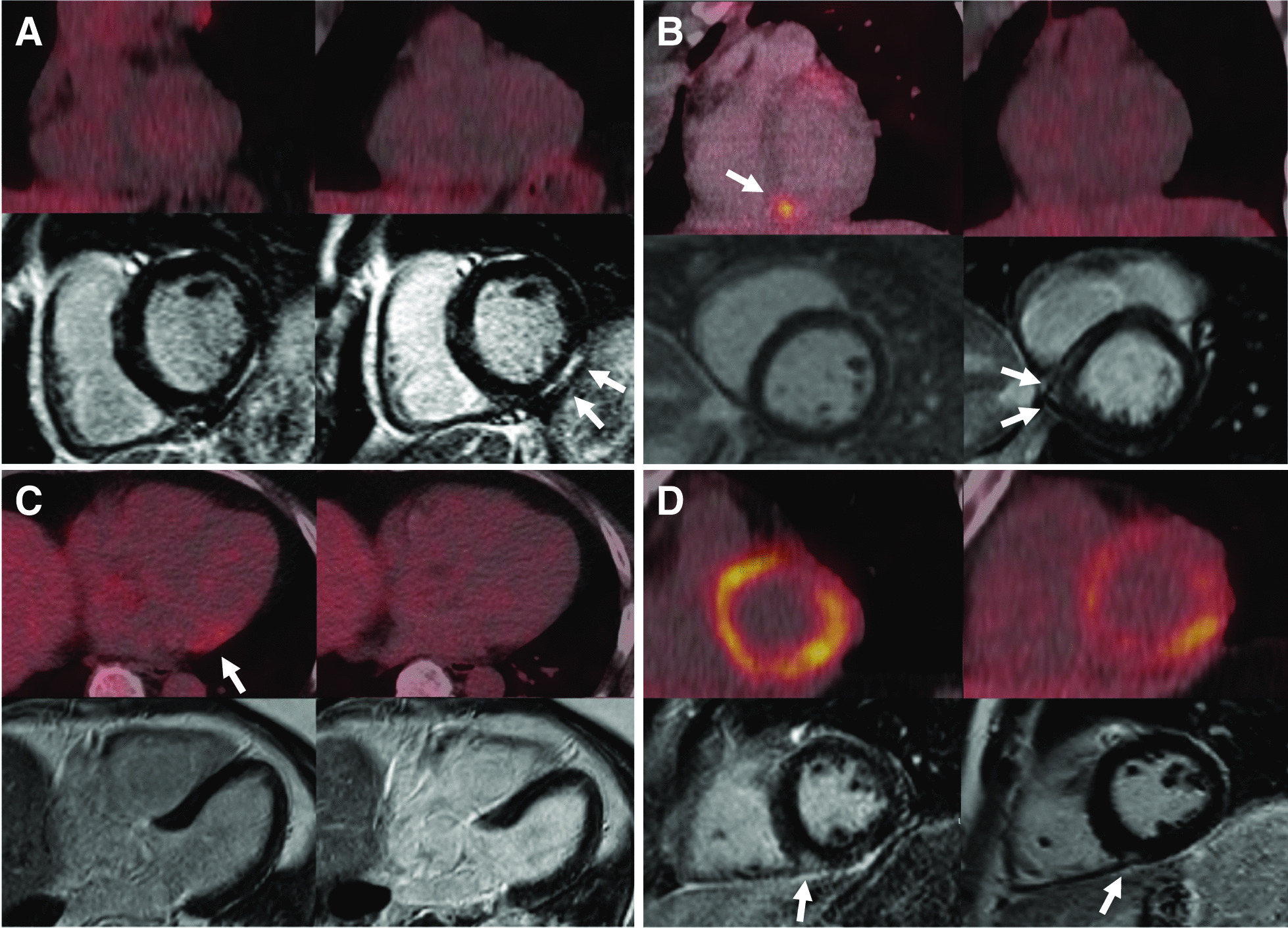
Examples of different FDG PET/CT and CMR patterns. In every image, baseline FDG PET/CT and CMR are shown on the left and repeated imaging on the right. **A** 48-year-old male patient who showed LGE uptake infero-lateral at first CMR (white arrows, short-axis view) without cardiac FDG-uptake (CMR+/PET−). The LGE increased at 2nd CMR with also increased T2-weighted signal (not shown); however, still no cardiac FDG-uptake was seen (CMR+/PET−), while the patient did not receive any immunosuppressive treatment. He was reclassified as probable CS. **B** A 36-year-old female patient who showed focal FDG-uptake infero-septal (white arrows) without LGE on CMR at baseline (CMR−/PET+). Between first and 2nd MDT, she was started on methotrexate 15 mg/week due to pulmonary sarcoidosis. Repeated imaging showed complete remission of cardiac FDG-uptake; however, CMR showed new LGE infero-septal (short-axis view, white arrows) and she was classified as CMR+/PET−. This patient was diagnosed with probable CS. **C** A 56-year-old male with focal FDG-uptake in the antero-lateral wall (white arrow, SUVmax 4.3) at baseline. He showed no LGE uptake on CMR (4 chamber view) and was classified as CMR−/PET+. The FDG-uptake was suspected to be physiologic and repeated imaging showed no cardiac FDG-uptake or LGE on CMR (CMR−/PET−). This patient received no immunosuppressive treatment between both MDT’s and CS was deemed “unlikely”. **D** A 47-year-old male patient who showed initial LGE inferoseptal on CMR (white arrow, short-axis view). However, after repeated imaging this LGE was interpreted as inferior hinge point fibrosis and not suspect for CS. Both FDG PET/CTs showed diffuse cardiac FDG-uptake (CMR+/PET+, CMR−/PET+). This patient did not receive any immunosuppressive therapies and was reclassified as “unlikely” CS

Examples of different CMR and FDG PET/CT patterns are shown in Fig. 3. After repeated imaging, LGE was present in nine patients (25.7%), while one patient showed increased T2-weighted signal, indicating myocardial oedema. This patient also showed an increased area of LGE compared to baseline, but without any myocardial FDG-uptake (Fig. 3A). Of the eleven patients classified as CMR+ at baseline, five were classified as CMR− after repeated imaging. In one patient this was due to inferior hinge point fibrosis, interpreted as innocent at repeated imaging and not suspect for CS (Fig. 3D). The remaining four patients initially all showed abnormalities, but at the 2nd CMR these abnormalities were absent and the findings at first CMR were interpreted as artefacts and not as LGE.

The presence of myocardial FDG uptake was seen in sixteen patients (45.7%) at re-evaluation, which was significantly lower compared to baseline (74.3%, *p* < 0.001). Additionally, the presence of a diffuse myocardial uptake pattern was seen in five (14.3%) versus ten patients (28.6%) at baseline (*p* = 0.13). A focal on diffuse or a focal pattern at re-evaluation was seen in four (11.4%) and seven patients (20.0%), respectively (Table 2). Focal myocardial FDG-uptake was seen in the antero-septal (*n* = 3), infero-septal (*n* = 1), infero-lateral (*n* = 3) and antero-lateral wall (*n* = 1). SUVmax was significantly higher at baseline compared to re-evaluation (median 4.2 vs 1.8, *p* < 0.01); however, as Table 2 shows, a higher proportion of patients showed no myocardial FDG-uptake at re-evaluation (54.3% vs 25.7%, *p* < 0.01). SUVmax of the focal or focal on diffuse myocardial FDG-uptake at baseline and re-evaluation was comparable (median 4.2 vs 4.0). Of the twenty-three patients who were initially classified as CMR−/PET+, three patients (13%) showed LGE on CMR after repeated imaging (Fig. 2). All three showed a focal myocardial FDG uptake pattern at initial FDG PET/CT and were reclassified as probable CS. Only one patient showed matching LGE and focal myocardial FDG-uptake (Fig. 3B). No patients with diffuse myocardial uptake on FDG PET/CT at initial imaging (CMR−/PET+) developed CMR abnormalities at re-evaluation.

When considering both imaging modalities together, the majority of patients (*n* = 19, 82.6%) with CMR−/PET+ at baseline, were reclassified as unlikely CS (*n* = 12, 52.2%) or remained diagnosed as possible CS (*n* = 7, 30.4%). On the contrary, four of the eleven (36.4%) patients with LGE presence at baseline were reclassified as probable CS (all CMR+/PET− at baseline). At follow-up, nine patients showed CMR abnormalities (both CMR+/PET+ and CMR+/PET−) of whom six (66.7%) were diagnosed as probable CS and three (33.3%) as possible CS. In eleven patients (31.4%) no imaging abnormalities were observed at follow-up, of whom eight (72.7%) were reclassified as unlikely CS.

### Impact of immunosuppressive therapies

At baseline, 14 patients were already on immunosuppressive therapies, all due to extracardiac sarcoidosis. Indications for immunosuppressive treatment were pulmonary- (*n* = 8) and neurosarcoidosis (*n* = 4) and sarcoidosis-related fatigue (*n* = 2). Used immunosuppressive therapies included prednisone monotherapy (*n* = 5), methotrexate monotherapy (*n* = 3), azathioprine monotherapy (*n* = 1) and prednisone and methotrexate combination therapy (*n* = 5). When comparing the patients with and without baseline therapy, QRS duration on electrocardiogram was the only significant different parameter (Additional file 1: Table S1). There were no differences in LVEF or FDG PET/CT results. Of the 14 treated patients, 11 were classified as CMR−/PET+ and three as CMR+/PET− at baseline (Fig. 2B). At the second MDT, the majority of this group was classified as either CMR−/PET+ or CMR−/PET− (*n* = 10, 71.4%). However, two patients showed new LGE on CMR at the second MDT (CMR+), but without myocardial FDG-uptake (PET−) and both were reclassified as probable CS. The 21 patients without baseline immunosuppressive treatment are shown in Fig. 2C. Of these, new treatment was started between both MDT’s in five patients due to pulmonary- (*n* = 4) or neurosarcoidosis (*n* = 1). Only in one of these five patients, FDG-uptake at baseline differed from follow-up (PET+ at baseline, PET− at baseline), but this patient also showed new LGE on CMR and was diagnosed with probable CS. Finally, Fig. 2D shows the remaining 16 treatment naïve patients who remained without therapy between both MDT’s. The proportion of patients with baseline FDG-uptake (PET+) or follow-up FDG-uptake (PET+) between this group (*n* = 16) and the treated group (*n* = 21) is comparable, 68.8% vs 78.9% (*p* = 0.70) and 43.8% vs 47.4% (*p* = 0.83), respectively.

### Secondary outcomes

After re-evaluation, immunosuppressive treatment was initiated in two patients who were reclassified as probable CS. Overall median LVEF was 60.0% [55.0–60.0] at baseline and 60.0% [51.0–61.0] at re-evaluation (*p* = 0.41). No patients showed a decrease in LVEF > 10%. Between both MDTs, one patient developed a third degree AVB despite discontinuation of beta-blockers. This was the patient who initially presented with a second-degree AVB while using beta-blockers. This patient was diagnosed with probable CS, despite the absence of imaging abnormalities at re-evaluation (CMR−/PET−). Another patient showed a second degree AVB during follow-up (CMR+/PET−, at both baseline and follow-up) and was also diagnosed as probable CS. Three patients showed a first degree AVB at baseline, which remained stable during follow-up. No other cardiac symptoms, ventricular arrhythmias or conduction disorders were observed. No patients died during follow-up. Serum markers (including CRP, ACE, NT-proBNP and sIL-2R) did not change significantly between both MDTs.

## Discussion

The purpose of this study was to evaluate the usefulness of repeated CMR and FDG PET/CT for the diagnosis of CS in patients initially diagnosed with possible CS. Most importantly, 25 patients (72%) could be reclassified as either probable (*n* = 9) or unlikely CS (*n* = 16). Furthermore, 3 out of 24 patients (13%) with an initially negative CMR but with myocardial FDG uptake, developed CMR abnormalities during follow-up and were diagnosed with probable CS. The clinical relevance of repeated imaging has to be investigated in future studies, since immunosuppressive treatment was initiated in only 6% of patients after re-evaluation. Nevertheless, clinical relevance does not only entail the change in treatment, since regular follow-up and prevention also prove to be valuable in patients with CS. Furthermore, rejecting a possible CS diagnosis can also prove valuable for the patient in terms of psychological uncertainty and follow-up burden.

To our knowledge, this is the first study to evaluate the usefulness of repeated CMR and FDG PET/CT for the diagnosis of CS in patients with possible CS diagnosis. In comparison to prior studies, all patients in our cohort routinely received both imaging modalities. We found that non-specific PET abnormalities rarely resulted in a probable CS diagnosis, as 83% of patients with initial CMR−/PET+ were re-evaluated as unlikely or possible CS. Several small studies have analysed findings of combined CMR and FDG PET/CT for the evaluation of CS but these studies showed mixed results [9–12]. Okune et al. performed a retrospective study and reported in a sub-analysis that two out of two patients (100%) with CMR−/PET+ were diagnosed as unlikely CS [9]. Similar results were reported by Soussan et al. [10], as they found that all three individuals with CMR−/PET+ out of a total of 35 included patients, were considered unlikely CS by the Japanese Ministry of Health and Welfare (JMHW) criteria [13]. On the contrary, a retrospective study by Vita et al. with 107 patients, reported that of eight patients with CMR−/PET+, four patients (50%) had probable or even highly probable CS [11]. Similarly, a study by Wicks et al. reported eleven patients with CMR−/PET+ of whom four patients (36.3%) were diagnosed with probable CS using the JMHW guidelines [12].

We found that patients with solely PET abnormalities were often reclassified as possible or unlikely CS at re-evaluation. However, three patients (13%) with initial CMR−/PET+ developed CMR abnormalities during follow-up and were reclassified as probable CS. All three patients showed focal myocardial FDG uptake at baseline. This emphasizes that, although not often, CMR−/PET+ can indicate early, active CS and precede CMR abnormalities. This is probably due to the presence of metabolically active inflammatory cells such as lymphocytes and macrophages in early stage CS. CMR with LGE is less sensitive in detecting this early inflammatory stage compared to FDG PET/CT [14]. Nevertheless, myocardial oedema as detected by increased T2-weighted signal can also prove valuable in detecting early stage CS [15]. Only one patient in our population showed myocardial oedema, but T2-weighted imaging was not consistently used. Remarkably, this patient did not show FDG-uptake and was not treated with immunosuppressive therapies. A possible explanation might be the 30 day time difference between repeated CMR and FDG PET/CT. An important finding was that none of the patients with diffuse FDG uptake developed abnormalities on CMR or were diagnosed with probable CS. This suggests that in patients with diffuse myocardial FDG uptake and normal CMR at initial evaluation it is sufficient to repeat only FDG PET/CT with an adequate dietary preparation. In patients with focal or focal on diffuse FDG uptake and an uncertain diagnosis, our data underline the importance of both repeated CMR and FDG PET/CT.

In our study, diffuse FDG uptake was classified as abnormal (PET+), whereas this is generally considered normal due to inadequate suppression of physiologic cardiac uptake [4, 16]. This might lead to differences in results compared to other studies, although Wicks et al. reviewed 51 patients with suspected CS undergoing hybrid FDG PET/CT and CMR, who were diagnosed using the JMHW guidelines [12, 13]. They compared annualized adverse event rates for patients with focal, focal on diffuse, diffuse and no myocardial FDG uptake. Remarkably, there was an event rate of 24% in patients with a diffuse uptake pattern versus 8% in patients with complete suppression of myocardial FDG uptake. Furthermore, they describe a patient with definite CS confirmed by endomyocardial biopsy, who had a diffuse uptake pattern on FDG PET/CT. This suggests that in some cases, diffuse myocardial uptake may represent CS and therefore cannot with certainty be classified as normal metabolism.

An important confounder in our study is that 40% of patients at baseline were already treated with immunosuppressive therapies. There were no clinically significant differences between both groups at baseline. Nonetheless, this treatment could have impacted FDG PET/CT results as earlier studies have shown that CS patients have reduction in cardiac FDG-uptake and lower cardiac SUVmax during treatment with immunosuppressive therapies [17–19]. However, in daily clinical practice clinicians will encounter sarcoidosis patients who are already treated with immunosuppressive treatment and in whom cardiac involvement is suspected. Our data show that when CS diagnosis is uncertain, repeated imaging with CMR and FDG PET/CT can also be valuable in this subpopulation. Furthermore, our population also included five patients who were newly started on immunosuppressive therapies for extra-cardiac sarcoidosis between both MDTs. Theoretically, myocardial inflammation could have been suppressed by these therapies; however, only one of these patients classified as PET+ at baseline was reclassified as PET−. This patient was still diagnosed as probable CS due to new LGE on CMR.

Our study had several limitations. Firstly, the modest sample size, which is a result of disease prevalence and supports the need for larger multicentre cohorts. Second, the retrospective character may lead to missing data or selection bias. Also, additional calculations like LGE as a percentage of left ventricular mass and heterogeneity of FDG uptake could not be evaluated in this study. Another limitation is that myocardial perfusion imaging was not performed in this study and could therefore not be used in the analysis. Also, our CMR studies did not consistently include T2-weighted sequences that could have detected myocardial oedema in the acute phases of CS. However, previous studies have shown that myocardial oedema was always accompanied with LGE [17, 18], while FDG PET/CT is considered a more sensitive imaging modality for acute inflammation [20]. Moreover, like all studies regarding the diagnosis of CS, this study is limited by the absence of a clinically functional reference standard. In our study the MDT discussion functioned as a reference standard and the MDT decision was based on a comprehensive clinical evaluation including laboratory tests, electrocardiogram, 24-h ambulatory heart rhythm monitoring and both CMR and FDG PET/CT. This approach is supported by other sarcoidosis expert centres [11, 21]. Finally, appropriate patient preparation prior to FDG administration is essential for achieving sufficient suppression of physiological myocardial glucose uptake to visualize inflammation. In our cohort, a large proportion of the FDG PET/CT scans showed a diffuse uptake pattern, considered as inadequate dietary preparation. This could have caused a high rate of false-positive FDG PET/CT scans, resulting in a high number of patients diagnosed with possible CS. A systematic review of Tang et al. concluded that the diagnostic accuracy improves after fasting for at least 12 h and a high fat low carbohydrate diet given at 3-6 h before imaging or heparin infusion [22]. A retrospective study from Sankaran et al. concluded that excellent myocardial FDG suppression can be achieved using a 24 h high fat very low carbohydrate diet and prolonged fasting [23]. Based on current literature, we recently changed the patient preparation instructions for FDG PET/CT. Patients are now instructed to have a carbohydrate-restricted diet for 24 h followed by a prolonged 12 h fasting period in order to reduce physiologic myocardial FDG uptake and decrease the need for repeated imaging.

## Conclusion

In conclusion, repeated CMR and FDG PET/CT may be useful in establishing or rejecting the diagnosis CS, when initial diagnosis is uncertain. Additional studies are required to determine the prognostic implications of repeated cardiac imaging for CS diagnosis as well as clinical relevance.

## Supplementary Information

**Additional file 1**. **Supplementary table 1.** Comparison of baseline characteristics and FDG PET/CT results between treated and treatment naïve patients at baseline.

## Data Availability

Data sharing is not applicable to this article as no datasets were generated or analysed during the current study.

## Abbreviations

AVB: Atrioventricular block
CMR: Cardiac magnetic resonance imaging
CS: Cardiac sarcoidosis
FDG PET/CT: Fluorodeoxyglucose positron emission tomography with computed tomography
JCS: Japanese Circulation Society
JMHW: Japanese Ministry of Health and Welfare
LGE: Late gadolinium enhancement
LVEF: Left ventricular ejection fraction
MDT: Multidisciplinary team
