# Supplementary material for: The usefulness of repeated CMR and FDG PET/CT in the diagnosis of patients with initial possible cardiac sarcoidosis
Source: EJNMMI Res. 2021 Dec 20;11:129. doi: 10.1186/s13550-021-00870-y (PMC8688603; doi:10.1186/s13550-021-00870-y)
Supplement: Supplementary file 1 — Additional file 1. Supplementary table 1. Comparison of baseline characteristics and FDG PET/CT results between treated and treatment naïve patients at baseline. [file 13550_2021_870_MOESM1_ESM.docx]

**Additional file 1: Table S1. Comparison of baseline characteristics and FDG PET/CT results between treated and treatment naïve patients at baseline**

| Variable | Patients without BL immunosuppressive treatment (n=21) | Patients with BL immunosuppressive treatment (n=14) | p-value |
| --- | --- | --- | --- |
| Age at diagnosis (years) | 54.0 ± 13.0 | 50.4 ± 12.4 | 0.43 |
| Male sex | 17 (81.0%) | 9 (64.3%) | 0.43 |
| Caucasian ethnicity | 19 (90.5%) | 13 (92.9%) | 1.00 |
| Body mass index (m^2^/kg) | 26.3 [24.7 – 29.4] | 28.4 [25.5 – 31.6] | 0.28 |
| NYHA functional class (I/II/III/IV) | 10 / 8 / 3 / 0 | 2 / 10 / 2/ 0 | 0.09 |
| Comorbidities   - Hypertension - Diabetes mellitus - Coronary artery disease | 7 (33.3%)  0 (0.0%)  1 (4.8%) | 2 (14.3%)  1 (7.1%)  0 (0.0%) | 0.26  0.40  0.40 |
| Extra cardiac sarcoidosis histologically or cytologically confirmed | 20 (95.2%) | 13 (92.9%) | 1.00 |
| Extra cardiac organ involvement   - Bilateral hilar lymphadenopathy - Pulmonary - Skin - Neurologic - Liver - Ocular | 19 (90.5%)  20 ((95.2%)  0 (0.0%)  1 (4.8%)  2 (9.5%)  3 (14.3%) | 10 (71.4%)  13 (92.9%)  1 (7.1%)  4 (28.6%)  1 (7.1%)  2 (14.3%) | 0.19  0.41  0.40  0.13  1.00  1.00 |
| Laboratory results   - CRP (mg/L) - NT-proBNP (pg/mL) (n=28) - ACE (U/L) - sIL-2R (pg/mL) | 2.5 [2.0 – 4.0]  39.5 [22.0 – 146.0]  46.0 [34.0 – 65.0]  4301 [2817 – 7365] | 3.0 [1.5 – 9.5]  56.5 [36.0 – 92.5]  44.0 [29.0 – 73.5]  3232 [2613 – 4385] | 0.50  0.49  0.65  0.17 |
| Electrocardiogram results   - Sinus rhythm - PQ-interval >200ms - QRS duration (ms) - Left bundle branch block - Right bundle branch block | (n=18)  17 (94.4%)  4 (22.2%)  100 [96.0 – 121.0]  0 (0.0%)  2 (11.1%) | (n=14)  14 (100%)  0 (0.0%)  94.5 [86.0 – 100.5]  0 (0.0%)  2 (14.3%) | 1.00  0.11  0.03  -  1.00 |
| Left ventricular ejection fraction (%) | 59.0 [54.0 – 61.0] | 60.0 [56.8 – 60.0] | 0.75 |
| Anti-arrhythmic drugs | 5 (23.8%) | 1 (7.1%) | 0.37 |
| ACE-inhibitors or ARBs | 8 (38.1%) | 3 (21.4%) | 0.46 |
| Myocardial FDG uptake pattern   - Focal - Focal on diffuse - Diffuse - None | 6 (28.6%)  3 (14.3%)  6 (28.6%)  6 (28.6%) | 4 (28.6%)  3 (21.4%)  4 (28.6%)  3 (21.4%) | 1.00  0.66  1.00  0.71 |
| Cardiac SUVmax at baseline | 4.2 [1.1 – 5.4] | 4.2 [3.1 – 6.2] | 0.63 |

*ACE = angiotensin-converting enzyme; ARB = angiotensin receptor blocker; BL = baseline; CRP = C-reactive protein; NYHA = New York Heart Association; sIL-2R: soluble interleukin-2 receptor; SUVmax: maximum standardized uptake value*
